# XX sex chromosome complement modulates immune responses to heat-killed Streptococcus pneumoniae immunization in a microbiome-dependent manner

**DOI:** 10.21203/rs.3.rs-3429829/v1

**Published:** 2023-11-02

**Authors:** Carly Amato-Menker, Quinn Hopen, Andrea Pettit, Jasleen Gandhi, Gangqing Hu, Rosana Schafer, Jennifer Franko

**Affiliations:** West Virginia University School of Dentistry; West Virginia University School of Medicine; West Virginia University School of Medicine; West Virginia University School of Medicine; West Virginia University School of Medicine; West Virginia University School of Medicine; West Virginia University School of Dentistry

**Keywords:** X chromosome, Four Core Genotype, gut microbiome, Kdm6a, short-chain fatty acids, sex differences, IgM, plasma cells, Streptococcus pneumoniae

## Abstract

**Background:**

Differences in male vs. female immune responses are well-documented and have significant clinical implications. While the immunomodulatory effects of sex hormones are well established, the contributions of sex chromosome complement (XX vs. XY) and gut microbiome diversity on immune sexual dimorphisms have only recently become appreciated. Here we investigate the individual and collaborative influences of sex chromosome complements and gut microbiome bacteria on humoral immune activation.

**Methods:**

Sham-operated and gonadectomized male and female Four Core Genotype (FCG) mice were immunized with heat-killed *Streptococcus pneumoniae* (HKSP). Humoral immune responses were assessed, and X-linked immune-related gene expression was evaluated to explain the identified XX-dependent phenotypes. *Ex vivo* studies investigated the functional role of *Kdm6a*, an X-linked epigenetic regulatory gene of interest, in mitogenic B cell activation. Additionally, we examined whether gut microbiome communities, or their metabolites, differentially influence immune cell activation in a sex chromosome-dependent manner. Endogenous gut microbiomes were antibiotically depleted and reconstituted with select short-chain fatty acid (SCFA)-producing bacteria prior to HKSP immunization and immune responses assessed.

**Results:**

XX mice exhibited higher HKSP-specific IgM-secreting B cells and plasma cell frequencies than XY mice, regardless of gonadal sex. Although *Kdm6a* was identified as an X-linked gene overexpressed in XX B cells, inhibition of its enzymatic activity did not affect mitogen-induced plasma cell differentiation or antibody production in a sex chromosome-dependent manner *ex vivo*. Enhanced humoral responses in XX vs. XY immunized FCG mice were eliminated after microbiome depletion, indicating that the microbiome contributes to the identified XX-dependent immune enhancement. Reconstituting microbiota-depleted mice with select SCFA-producing bacteria increased humoral responses in XX, but not XY, FCG mice. This XX-dependent enhancement appears to be independent of SCFA production in males, while female XX-dependent responses relied on SCFAs.

**Conclusions:**

FCG mice have been used to assess the influence of sex hormones and sex chromosome complements on various sexually dimorphic traits. The current study indicates that the gut microbiome impacts humoral responses in an XX-dependent manner, suggesting that the collaborative influence of gut bacteria and other sex-specific factors should be considered when interpreting data aimed at delineating the mechanisms that promote sexual dimorphism.

## Plain English Summary

Male and female immune systems differ in their ability to respond to infectious challenge. While males tend to be more susceptible to infection and produce lower amounts of antibodies in response to vaccination, females are more prone to develop autoimmune and inflammatory diseases. Key contributors to these differences include sex hormones, sex chromosome complement (XX in females vs. XY in males), and distinct gut microbial communities capable of regulating immune activation. While each factor has been studied individually, this research underscores the potential for these factors to collaboratively impact immune activation. Here, possession of an XX vs. XY sex chromosome complement was demonstrated to enhance antibody responses to heat-killed *Streptococcus* pneumoniae vaccination. While attempting to determine the underlying cause of this immune enhancement, the gut microbiome was identified to play a critical role. In the absence of an intact gut microbiome, XX immune activation was reduced to levels similar to those seen in XY sex chromosome complement-possessing mice. Replacement of the depleted gut microbiomes with select bacterial species rescued the XX-dependent immune enhancement. Further studies are needed to determine exactly how these microbial communities impact immune activation in a sex chromosome complement-dependent manner. Our findings highlight the need to consider the collaborative effects of individual sex-specific factors when attempting to understand immune sex biases, as a better understanding of these interactions will likely pave the way for improving therapeutics and vaccines tailored to both sexes.

## Background

Sex differences in immune responses have been well characterized (1). In general, females elicit stronger humoral and cell-mediated responses to infection and respond better to vaccination than males, but in turn are more susceptible to autoimmune and inflammatory disorders (1–7). Historically, hormonal differences have been described as the predominant determinant contributing to such sexual dimorphisms, as estradiol and progesterone are known to be immunostimulatory in nature and testosterone immunosuppressive (8–10). However, the prevalence of immune sex differences before puberty and following the onset of menopause suggests a role for non-hormonal factors as well (1, 11–14).

Recently, the influence of the XX vs. XY sex chromosome complement on immune cell activation has become increasingly appreciated. The X chromosome encodes for a large number of immune-related genes, as well as epigenetic regulators associated with lymphocyte activation and differentiation (15). While the dosages of X-linked genes are typically balanced between males and females via the process of X chromosome inactivation (XCI) (16), X-linked genes related to immunity have been demonstrated to more readily escape XCI in immune cells than other somatic cell types, and are uniquely regulated in B lymphocytes (17–21). Multiple X-linked immune-related genes have been demonstrated to escape inactivation and their biallelic expression correlates with disease. For example, *CD40L and CXCR3* are biallelically expressed in T cells isolated from female, but not male, systemic lupus erythematosus (SLE) patients (22, 23). *TLR7* and *TLR8* have also been identified as escape genes and are overexpressed in primary B lymphocytes, monocytes, and plasmacytoid dendritic cells isolated from female SLE patients and XXY Klinefelter syndrome males (20, 24). In addition to SLE, sex chromosome complement-dependent influences have been identified in animal models of experimental autoimmune encephalitis (EAE) (25), anti-viral immunity (26), stroke-induced neuroinflammation (27, 28), and various metabolic disorders (29).

Sex has also been shown to influence the complexity and diversity of gut microbiome populations (30–33). Using a panel of over 100 diverse inbred strains of mice, Org *et al*. identified distinct gut microbial communities in male vs. female mice whose composition was influenced by the presence of male vs. female sex hormones (31). Reciprocally, distinct sex-specific gut microbial communities have also been demonstrated to modulate sex steroid production and influence immune cell activation by direct and indirect mechanisms. Differences in male vs. female susceptibility to type I diabetes have been directly linked to sex-specific gut microbiome populations capable of enhancing systemic androgen concentrations in male mice, or in female mice colonized with adoptively transferred male gut microbial communities (30, 32). While these studies demonstrate that sex hormones and gut microbial communities collaborate to influence immune activation, to our knowledge no previous study has evaluated whether sex chromosome-dependent immune phenotypes are influenced by the gut microbiome.

Previously, our lab demonstrated that the immunomodulatory compound propanil (3,4-dichloropropionanalide) enhances immune responses to heat-killed *Streptococcus pneumoniae* (HKSP) immunization in an XX sex chromosome complement-dependent manner using Four Core Genotype (FCG) mice (34). FCG mice exhibit one of four different genotypes: XX and XY females (ovaries) and XX and XY males (testes) and allow for the study of individual and collaborative sex hormone and sex chromosome complement-dependent effects (35–38). While the mechanism mediating this propanil-mediated XX-dependent immune enhancement has yet to be defined, the contribution of circulating sex hormones was ruled out, as gonadectomy did not inhibit propanil-mediated immune enhancement (34). Interestingly, *in vivo*, propanil is broken down into two major metabolites, 3,4-dichloroanaline (DCA) and the short-chain fatty acid (SCFA) propionate, via hepatic acylamidase-mediated hydrolysis (39). Given that propionate, a SCFA with known immunomodulatory function, is also one of the major metabolic end-products of dietary fiber fermentation by gut microbiome bacteria (40–47), we hypothesized that the gut microbiome and/or its metabolic byproducts may collaboratively influence sex-specific immune outcomes in a sex chromosome-dependent manner.

In the following study, we evaluated the independent and collaborative influences of the XX vs. XY sex chromosome complements and sex-specific gut microbial communities on immune activation. Following immunization with HKSP, enhanced HKSP-specific antibody secreting cells and plasma cell differentiation were noted in XX vs. XY male and female FCG mice. While *Kdm6a*, an X-linked epigenetic regulator with known immunomodulatory function, was demonstrated to escape X chromosome inactivation and to be overexpressed in XX-possessing cells, the ability of *Kdm6a* to promote plasma cell differentiation or antibody production was not identified to be sex chromosome-dependent. This suggested that other XX-dependent regulatory factors must be contributing to the XX-dependent phenotype. Interestingly, and importantly for the interpretation of other studies evaluating sex chromosome-dependent effects, antibiotic depletion of the endogenous microbiome reduced humoral responses to HKSP immunization in XX mice to levels similar to XY mice, but had no influence on XY responses. This demonstrates that stronger immune responses in XX animals are microbiome-dependent. Given that the sex chromosome complement did not alter microbiome compositions, additional studies are warranted to evaluate the mechanism by which these microbes mediate their sex chromosome-dependent effect.

## Materials and Methods

### Four Core Genotype model

B6.Cg-Tg(Sry)2Ei *Sry*^*dl1Rlb*^/ArnoJ (XY^−^ *Sry*) male mice were originally purchased from The Jackson Laboratory (Bar Harbor, Maine). A colony was subsequently maintained at West Virginia University by breeding B6.Cg-Tg(Sry)2Ei *Sry*^*dl1Rlb*^/ArnoJ (XY^−^ *Sry*) males with C57BL/6J females (The Jackson Laboratory). All purchased animals were allowed to acclimate for one week prior to use. The sex determining region of the Y chromosome, the *Sry* gene, had previously been deleted from the Y chromosome of XY^−^ *Sry* mice and inserted as a transgene onto autosome 3. Breeding of XY^−^ *Sry* male mice with wildtype C57BL/6 female mice produced FCG mice: XX or XY gonadal females (XXF and XYF) and XX or XY gonadal males (XXM and XYM), as shown in Supplemental Fig. 1. FCG mice were weaned at 21 days of age. The genotype of the offspring was determined by PCR amplification of the following genes: *Sry, Ymt,* and *Myo*, using DNA isolated from tail samples or ear punches obtained at weaning. QIAGEN Fast Cycling PCR kit (Qiagen, Louisville, KY) was used for PCR amplification.

Mice were housed in microisolator cages in specific pathogen-free conditions on a 12 hr light–dark cycle with food and water provided *ad libitum*. Studies were conducted in accordance with all federal and institutional guidelines for animal use and were approved by the WVU Institutional Animal Care and Use Committee, protocol #1603001079

#### Preparation of heat-killed Streptococcus pneumoniae (HKSP) and immunization

*S. pneumoniae* strain R36A, an avirulent, nonencapsulated strain, was grown to mid-log phase in Todd-Hewitt broth + 1% yeast extract (Becton Dickinson, Sparks, MD) and stored at − 80°C. For immunization, stock was cultured in a candle jar for 18 hours at 37°C on blood agar plates (Becton Dickinson). Colonies were selected and suspended in 200 ml broth, grown at 37°C to an absorbance reading at 600nm of 0.4 and heat-killed for 1 hour in a 60°C water bath. A final concentration of 10^9^ CFU/mL was established in PBS based on colony counts. Sterility was confirmed by culture and heat-killed *S. pneumoniae* stored at − 20°C. Mice were immunized intraperitoneally with 2×10^8^ CFU HKSP, which elicits an optimal PC-specific antibody response 7 days post-vaccination (48, 49).

### Collection of samples for immunologic assessments

Mice were euthanized with 100 μl Euthasol (50 mg/ml, Virbac Inc., Fort Worth, TX) 7 days following immunization. Serum was collected by cardiac puncture. To generate single cell splenocyte suspensions, spleens were dissociated through 70μM cell strainers (Thermo Fisher, Florence, KY) in RPMI-1640 (Corning, Manassas, VA), 10% heat inactivated fetal bovine serum (FBS, Hyclone Laboratories, Inc, Logan, UT), 10 mM HEPES (Sigma-Aldrich, Burlington, MA), 1 mM L-glutamine (Gibco, Rockville, MD), 5×10^− 5^M 2-mercaptoethanol (Sigma-Aldrich), 100 U/ml penicillin (Gibco), and 100 μg/ml streptomycin (Gibco). Red blood cells were lysed with Tris-buffered ammonium chloride. Cell suspensions were washed and counted using a hemacytometer. Viability was determined using Trypan blue dye exclusion (Sigma-Aldrich). When indicated, B cells were isolated from splenocytes by negative selection with the EasySep^™^ mouse B cell isolation kit (STEMCELL Technologies, Kent, WA). RNA was isolated from splenocytes or isolated B cells via Trizol:chloroform extraction or by the RNeasy Protect Mini Kit (Qiagen, Valencia, CA). Contaminating genomic DNA was eliminated using the Invitrogen TURBO DNA-*free*^™^ Kit (Thermo Fisher).

### Measurement of antibody-secreting cells (ASCs)

Millipore MultiScreen^®^ 96-well filter plates (Sigma-Aldrich) were coated with 50μl phosphorylcholine (PC)-BSA (Biosearch Technologies, Petaluma, CA; 10 μg/ml) overnight at 4°C. In subsequent steps, plates were washed with PBS + 0.01% Tween-20. Plates were blocked with 200 μl/well RPMI medium + 25% FBS for 2 hours at 37°C. Plates were washed and splenocytes (100 μl/well) added at 5×10^6^ cells/ml and 1×10^6^ cells/ml, each plated in triplicate. Plates were incubated for 4–6 hours at 37°C 5% CO_2_. After washing, goat anti-mouse alkaline phosphatase (AP) conjugated IgM antibody (Southern Biotechnology Associates, Birmingham, AL), diluted 1/2000 in PBS + 1% BSA + 0.05% Tween-20, were added to the appropriate wells (100 μl/well). Plates were incubated overnight at 4°C and washed. Phosphatase substrate tablets (Sigma-Aldrich) were dissolved in water and 100 μl added to each well. Color development was stopped by washing with water. The number of spots/well was counted using a dissection microscope (ZEISS, Dublin, CA). The number of ASC was calculated using the mean number of spots from triplicate wells. Mice demonstrating an average of less than 20 spots in the 5×10^6^ dilution were considered non-responders and not included in subsequent analyses. The number of ASC was normalized to 1×10^6^ splenic B220 + B cells as determined by flow cytometric analysis.

### Flow cytometry

The Fc receptor of 200,000 cells were blocked with ChromPure IgG (Jackson ImmunoResearch, West Grove, PA) for 20 minutes, washed, and then stained with the following antibodies for 25 minutes on ice in the dark: rat anti-mouse B220-APC (RA3–6B2; BD Biosciences, San Diego, CA) and CD138-BV786 (281–2; BD). After staining, cells were washed and fixed in 0.04% paraformaldehyde (Thermo Fisher). Live cells were determined utilizing a Live/Dead Fixable Yellow Dead Cell Stain Kit (Invitrogen, Carlsbad, CA), and where applicable absolute cell number was determined using AccuCount beads (Spherotech, Lake Forest, IL). For each sample, 10,000–30,000 cells were collected for analysis (FCS Express software) on an LSRFortessa (BD).

### Gonadectomy surgeries

Bilateral castration or ovariectomy was performed on eight- to twelve-week-old mice by standard procedure (50). Briefly, mice were anesthetized with isoflurane. Incisions were made through the skin and the underlying abdominal wall. The testes or ovaries were isolated and heated forceps used to cauterize the vas deferens and the blood vessel or transect the tip of the uterine horn and cauterize the blood vessels. The abdominal wall was closed with a suture and skin incisions closed with wound clips. Sham-operated mice (Sham) underwent the same procedure, but the testes or ovaries were left intact. Gonadectomized (Gdx) and Sham mice were housed four to five weeks following surgery before being used in experiments.

### Measurement of antibody concentrations by ELISA

Immulon 2 plates (ThermoLabsystems, Pittsburgh, PA) were coated overnight at 4°C with goat anti-mouse human adsorbed unlabeled IgM (Southern Biotech; 100 μl/well). Plates were washed, blocked with 3% BSA in PBS at 37°C overnight, washed, and 100μl/well of four two-fold dilutions of sera in PBS + 1% BSA were added starting at 1:2. Sample containing plates were incubated for 1 hour at 37°C and washed. Goat anti-mouse AP conjugated antibodies (Southern Biotech; 100μl/well) were added for 1 hour at 37°C. Plates were washed and 100μl of phosphatase substrate tablets (Sigma-Aldrich) dissolved in p-Nitrophenyl Phosphate, Disodium Salt (PNPP) buffer was added to wells. Absorbance was read at 405nm on an xMark^™^ Microplate Spectrophotometer with the Microplate Manager^™^ Software (Bio-Rad, Hercules, CA). Standard curves were generated using serial dilutions of purified rat anti-mouse IgM (Clone II/41, BD), and the 4-parameter fit equation used to calculate sample concentrations.

### RNA Sequencing and analysis

After quantification and quality assessment of splenocyte RNA, 500ng of total RNA was used to prepare Illumina-compatible libraries using the KAPA stranded mRNA library kit (Kapa Biosystems, Wilmington, MA). Sequencing was performed as 2 × 51 cycles on an Illumina HiSeq 2000 (Marshall Genomics Core). RNA-Seq data analysis followed previously described procedures (51, 52). Briefly, RNA-Seq short reads were aligned to the mm10 with subread (53). Read counts against RNA-Seq gene annotation was summarized with FeatureCounts (54). Differentially expressed genes were predicted by EdgeR with FDR less than 0.1 and a log_2_FC more than 0.585. Gene expression values (RPKM; log_2_) across groups were visualized with GraphPad Prism version 9 for Windows (La Jolla, CA, www.graphpad.com).

### qRT-PCR

RNA concentrations and purity were measured on a Nanodrop 2000 (Thermo Fisher). cDNA was synthesized with the GoScript^™^ Reverse Transcription kit (Promega, Madison, WI). Transcripts were amplified by qRT-PCR using the primers below (Life Technologies, Carlsbad, CA) and the incorporation of PowerUp^™^ SYBR^™^ Green Master Mix (Life Technologies) was measured on the StepOnePlus RT-PCR system (Applied Biosystems, Foster City, CA) to determine expression levels. The following cycling conditions were utilized: 95°C for 2 min, 40 cycles of (95°C for 15s -- 60°C for 1 minute), 95°C for 15s. *Kdm6a* expression was determined by normalization to *Gapdh* expression.

### Western Blots

Total protein was isolated from HKSP-immunized mouse splenocytes using m-PER^™^ Mammalian Protein Extraction Reagent (Thermo Fisher) with 1% protease inhibitor (Cell Signaling, Danvers, MA). Protein concentrations were quantified using the Pierce^™^ Coomassie Plus (Bradford) Assay Kit (Thermo Fisher). Equal amounts of protein were boiled for 5 minutes and then resolved by SDS-Page on pre-cast Bolt 4–12% Bis-Tris Plus gels at 100V for 60–120 minutes. The gel was electrophoretically transferred to polyvinylidene fluoride membranes using the iBlot^™^ 2 Transfer system (Invitrogen). Following transfer, membranes were blocked with 5% milk for one hour, followed by staining with primary antibody (anti-kdm6a 1:1000 and anti-β-tubulin 1:1000, Abcam, diluted in 5% milk) and incubated overnight. Membranes were then washed, incubated with secondary antibody (HRP anti-rabbit IgG) for one hour, then visualized using the SuperSignal West Pico PLUS substrate (Thermo Fisher). Blots were imaged on an iBright CL1500 (Invitrogen) imaging system and quantified using ImageJ with normalization to β-tubulin.

### RNA-FISH

Biallelic expression of *Kdm6a* in B cells was observed using the Stellaris^®^ RNA FISH system (Biosearch Technologies). Briefly, B cells from HKSP-immunized mouse spleens were purified by negative selection with the EasySep^™^ mouse B cell isolation kit (STEMCELL Technologies). 5×10^6^ B cells were washed with PBS and resuspended in 1mL fixation buffer. After 10 minutes at room temperature, cells were washed three times with 1X PBS and then permeabilized in 1mL of 70% ethanol for at least one hour at 4°C. Cells were washed and resuspended in hybridization buffer containing the *Xist* (mouse *Xist* with Quasar^®^ 570 Dye, Biosearch Technologies) and/or *Kdm6a* (custom from Biosearch Technologies with Quasar^®^ 670; sequences available upon request) probes and incubated at 37°C overnight in the dark. Cells were washed thoroughly and resuspended in 30μL of ProLong^™^ Glass Antifade Mountant with NucBlue^™^ (Invitrogen) and 5–10μl mounted on *Superfrost Plus* microscope slides (Thermo Fisher). 2D images and Z-stacks were acquired on an inverted Nikon TI-E microscope with an A1R dual Galvano/resonant scanning confocal system equipped with four lasers (405 nm, 488 nm, 561 nm, 640 nm) and analyzed with NIS-Elements Advanced Research. For each sample, five sections were imaged and the following quantified: total number of cells, number of cells with an *Xist* cloud, and number of cells with *Xist* and *Kdm6a* colocalization.

#### Ex vivo splenocyte stimulation

Splenocytes isolated from naïve mice were cultured at 0.5×10^6^ cells/mL in 12-well tissue culture plates using the following culture media: RPMI-1640 (Corning), 10% heat inactivated fetal bovine serum (FBS, Hyclone Laboratories), 10 mM HEPES (Sigma-Aldrich), 1 mM L-glutamine (Gibco), 5×10^− 5^ M 2-mercaptoethanol (Sigma-Aldrich), 100 U/ml penicillin (Gibco), and 100 μg/ml streptomycin (Gibco). Cells were stimulated using LPS (5μl/mL, *E. coli* O55:B5; Sigma-Aldrich) and recombinant mouse IL-4 (0.1μg/mL, R&D Systems, Minneapolis, MN). To study the effects of KDM6a inhibition, cells were incubated with 0.25, 0.5, or 2.0μM GSK J4 or GSK J5 (R&D Systems) reconstituted in DMSO or with DMSO alone for 30 minutes prior to stimulation. To examine the impact of SCFA on plasma cell differentiation, cells were exposed to propionate dissolved in culture media (0.5, 1, and 2mM; Sigma-Aldrich) 30 minutes prior to stimulation with LPS and IL-4. All cell cultures were supplemented with LPS and IL-4 (at half concentration of initial stimulation) every 24 hours during the experiment.

### Microbiome Assessment

Total DNA was extracted from fecal samples using the DNeasy PowerSoil DNA isolation kit (Qiagen) according to the manufacturer’s recommended protocol. PCR amplification of the V3 region of the 16sRNA RNA gene was performed by utilizing high pressure liquid chromatography-purified primers (Integrated DNA Technologies; Coralville, IA), AccuPrime PCR Kit (Invitrogen) and cycling conditions previously described by Fadrosh *et al*. (55). Briefly, cycling conditions included: 95°C for 6 minutes denature; 95°C for 2 minutes, 50°C for 2 minutes, 72°C for 2 minutes 30 cycles; 72°C for 4 minutes extend. Each reaction contained 0.5 μl Taq polymerase, 5 μl 10x buffer 1(600 mM Tris-SO4 (pH 8.9), 180 mM (NH4)2SO4, 20 mM MgSO4, 2 mM dGTP, 2 mM dATP, 2 mM dTTP, 2 mM dCTP, thermostable AccuPrime^™^ protein, 10% glycerol), 20 μM forward primer, 20 μM reverse primer, and up to 60 ng DNA in a total volume of 50 μl. Primer sequences are available upon request. Following quantitation and quality control analysis of the amplified 16s rRNA amplification product, paired-end sequencing (2 × 150 bp) was performed using the Illumina MiSeq located in the Genomics Core Facility at WVU.

Microbiome sequencing files were analyzed using QIIME2 (version 2020.11) (56, 57). Sequencing quality was inspected using fastQC (58). DADA2 (59) was used to optimize the parameter for quality control and read trimming. Taxonomy assignments were performed using the SILVA 132 (60) database at 97% identities. Rarefaction curve analysis on alpha diversity was used to estimate the sampling completeness and for OTU calculations. Beta diversity metrics calculated included Jaccard distances, unweighted UniFrac distances, weighted UniFrac distances, and generalized UniFrac distances. Significance in the difference between alpha and beta diversities was based on Kruskal–Wallis test and permutational multivariate analysis of variance, respectively.

### Short-chain fatty acid concentrations

Fecal samples were collected on dry-ice and stored at −80°C until analysis. Concentrations of eight short-chain fatty acids: acetic acid (C2, acetate), propionic acid (C3, propionate), isobutyric acid (C4), butyric acid (C4, butyrate), 2-methyl-butyric acid (C5), isovaleric acid (C5), valeric acid (C5) and caproic acid (hexanoic acid, C6) were assessed by LC-MS/MS (Metabolon, Morrisville, NC), using their Metabolon Method TAM135: “LC-MS/MS Method for the Quantitation of Short Chain Fatty Acid (C2 to C6) in Human Feces” workflow.

### Microbiome depletion

Endogenous gut microbiomes were depleted using an antibiotic cocktail containing metronidazole (10 mg/ml, Sigma-Aldrich), vancomycin (10 mg/ml, Sigma-Aldrich), neomycin (20 mg/ml, Sigma-Aldrich) and ampicillin (20 mg/ml, VWR, Radnor, PA) in sterile water. The antibiotic cocktail (100 μl) was administered via oral gavage every day for 3 days and then every other day until the end of the experiment. Controls received sterile water alone. Chow was removed from all cages 4 hours prior to antibiotic or water gavage to optimize antibiotic absorption. Microbiome depletion was verified using the LIVE/DEAD^®^ BacLight^™^ Bacterial Viability and Counting Kit (Life Technologies) and subsequent acquisition on the LSRFortessa flow cytometer (BD). For all experiments using antibiotics, mice were housed by group to eliminate cross-contamination from fecal ingestion, provided with autoclaved drinking water and irradiated chow throughout the experiment, and provided with clean autoclaved cages after each antibiotic treatment.

### Culture of SCFA-Producing Bacteria

The bacterial strains *Bifidobacterium longum, Clostridium symbiosum*, and *Lactobacillus fermentum* were purchased from ATCC. All strains were cultured in brain heart infusion (BHI) medium (Sigma-Aldrich). *L. fermentum* was cultured in aerobic conditions, while *B. longum* and *C. symbiosum* were cultured anaerobically using anaerobic gas jars, EZ gas packs (BD), and pre-reduced media. Under sterile conditions, bacteria were inoculated in 5–6mL culture medium and incubated at 37°C for two days. Secondary inoculations were done inoculating 1mL of the initial culture into 5–6mL of fresh culture medium. Cultures were then allowed to incubate at 37°C for 1–2 additional days. Once OD values reached at least 0.8, as measured on the xMark^™^ Microplate spectrophotometer (Bio-Rad), serial dilutions were made and their ODs measured. Dilutions were then plated on pre-reduced Brucella Agar with 5% sheep blood plates (*B. longum* and *C. symbiosum*; Anaerobe Systems, Morgan Hill, CA) or Blood Agar (*L. fermentum*; TSA with sheep blood, Remel, Lenexa, KS) plates and incubated overnight at 37°C. CFUs were counted and growth curves generated to establish standard curves for each bacterial species. Bacteria were then frozen in pre-reduced glycerol and stored at −80°C for future use. Fresh cultures were initiated from frozen stocks 6 days prior to the first day they were needed in an experiment, with new inoculations into fresh media every other day. DNA was isolated from individual bacterial colonies and amplified by PCR using 16s primers (Eurofins Genomics, Louisville, KY). Sequencing of amplified PCR products allowed for comparison of sequences to known BLAST database for species confirmation. 16s primer sequences were as follows: Forward: 5’-CGG TTA CCT TGT TAC GAC TT-3’. Reverse: 5’-AGA GTT TGA TCC TGG CTC AG-3’.

### Reconstitution of SCFA-Producing Bacteria and Inulin Administration

The endogenous microbiome was depleted in all mice by antibiotic gavage as described above, with mice receiving antibiotics daily for 3 days. To reconstitute the microbiome with SCFA-producing bacteria, mice received a cocktail containing the following bacteria: *B. longum* (1×10^7^), *C. symbiosum* (5×10^6^), and *L. fermentum* (1×10^9^) via oral gavage on Days 4 and 5. Bacterial counts were determined by OD measurements at 600nm and previously established standard growth curves. Control mice received oral gavage of sterile medium alone. Inulin (MilliporeSigma) was diluted in sterile water and provided as a second oral gavage (10mg in 100μl) on Days 4 and 5. Mice not receiving inulin were provided a second oral gavage of sterile water (100μl) alone. All mice were then immunized (i.p.) with 2×10^8^ CFU HKSP on day 6. Mice receiving inulin alone continued to receive antibiotics in sterile water gavage every other day throughout the experiment, while the experimental groups received oral gavage of sterile water alone. Fecal pellets were collected at Day 0 (pre-antibiotics), Day 4 (post-antibiotics and pre-bacteria +/− inulin gavage), Day 6 (post-bacteria +/− inulin, pre-HKSP immunization), and Day 13 (euthanasia) and assessed for gut colonization status by the LIVE/DEAD^®^ BacLight^™^ Bacterial Viability and Counting Kit (Life Technologies). SCFA levels were assessed by LC-MS/MS (Metabolon, Morrisville, NC).

### Statistics

**Figure 1:**
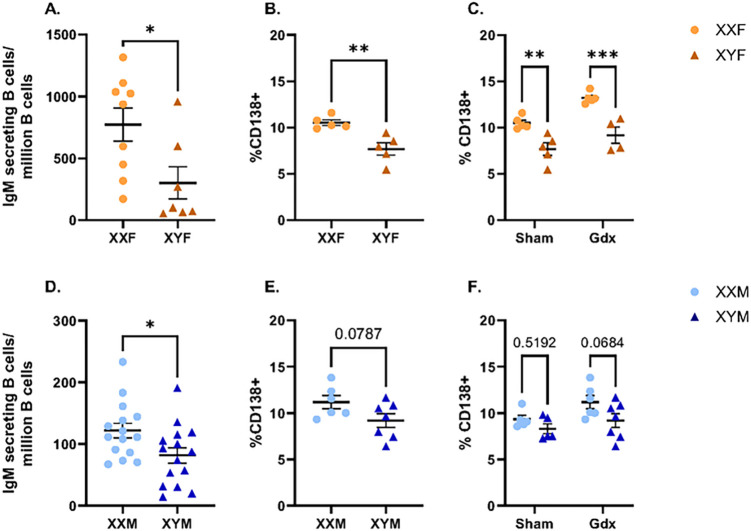
Possession of an XX vs. XY sex chromosome complement influences humoral responses to HKSP immunization. Immune responses against HKSP were assessed in FCG females (A-C) and males (D-F) one-week post-HKSP immunization. Numbers of HKSP-specific IgM-secreting B cells (A, D) and percentages of CD138+ plasma cells (B, E) were measured using ELSPOT and flow cytometry, respectively. To assess the impact of sex hormones on sex chromosome-dependent phenotypes, percentages of CD138+ plasma cells were assessed in sham-operated vs. gonadectomized female (C) and male (F) FCG mice following the same immunization protocol. Data are represented as the mean +/− SEM with each data point representing one mouse. Unpaired t-tests (A-B, D-E) or two-way ANOVA followed by Sidak’s multiple comparisons test (C, F). *p<0.05; **p<0.01; ***p<0.001. Sham = sham-operated; Gdx = gonadectomized.

**Figure 2:**
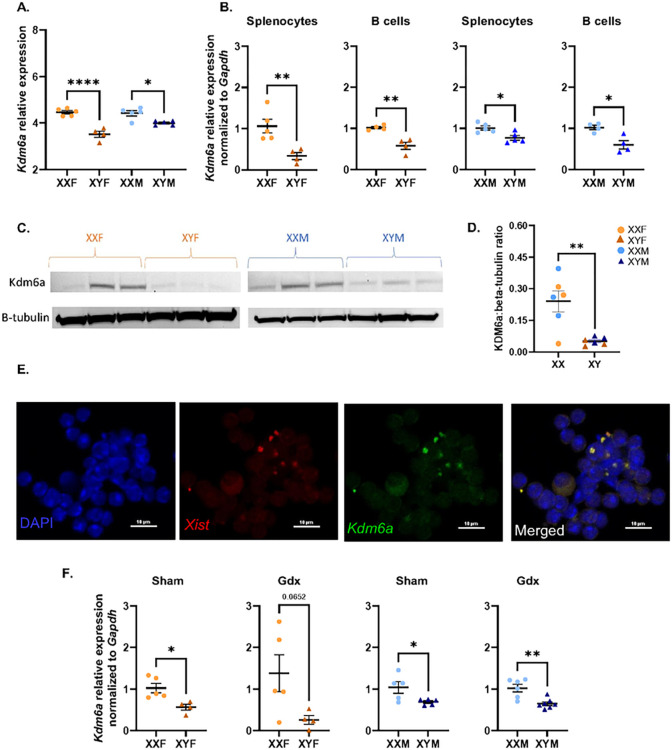
Kdm6a is differentially expressed in XX vs. XY splenocytes and B cells isolated from HKSP-immunized FCG mice. One-week post-HKSP immunization, RNA-Sequencing was performed on splenocytes isolated from male and female FCG mice. Levels of Kdm6a expression are represented as relative expression using EdgeR values (A). Differential expression of *Kdm6a* was confirmed by qRT-PCR using both splenocytes and B cells isolated from HKSP-immunized FCG mice (B). KDM6a protein levels were assessed by western blot using lysates generated from HKSP-immunized FCG splenocytes (C, D). Relative levels of protein expression were quantified as KDM6a:beta-tubulin ratios (D). RNA-FISH was utilized to determine if *Kdm6a* escaped XCI in B cells isolated from female XX FCG mice previously immunized with HKSP. Representative images of individual DAPI, *Xist*, and *Kdm6a*, as well as merged images, are presented (E). Colocalization of *Kdm6a* and *Xist* signals was considered indicative of XCI escape (E). *Kdm6a* expression was also assessed in B cells from HKSP-immunized gonadectomized and sham-operated animals (F). Data are represented as the mean +/− SEM with each data point representing one mouse. Statistics by one-way ANOVA followed by Tukey’s multiple comparisons test (A) or unpaired t-tests (B, D, F). *p<0.05; **p<0.01; ****p<0.0001. Sham = sham-operated; Gdx = gonadectomized.

**Figure 3:**
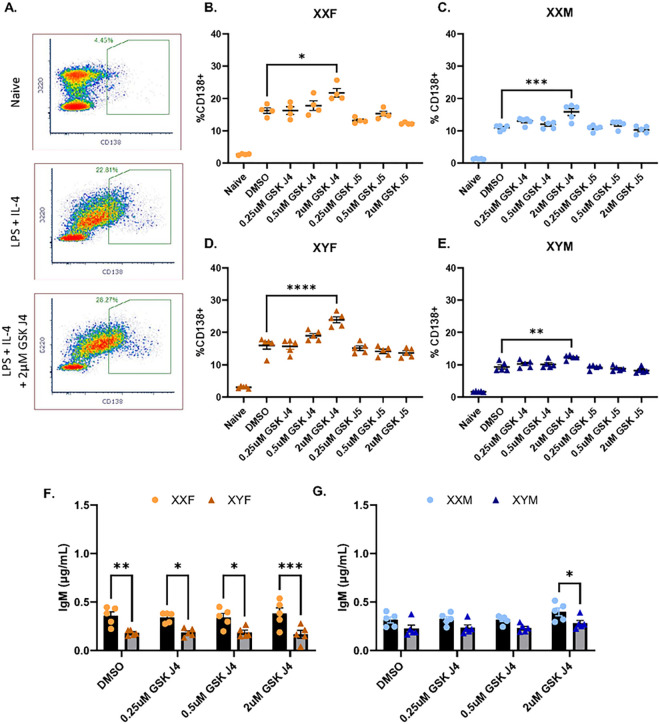
KDM6a inhibition enhances plasma cell differentiation, but not IgM secretion, similarly in all four genotypes. Splenocytes isolated from naïve FCG mice were stimulated *ex vivo* with IL-4 (0.01μg/mL) and LPS (5μg/mL) in the presence or absence of GSK J4 or its inactive isomer GSK J5. Representative density plots of flow cytometric data from naïve (day 0) or stimulated splenocytes (day 3) +/− GSKJ4 exposure are depicted in (A). Percentages of CD138+ plasma cells in XXF (B) and XYF (D) females and XXM (C) and XYM (E) male FCG mice were quantified by flow cytometry. Supernatants were collected and total IgM concentrations were assessed by ELISA for each stimulation condition (F-G, Suppl. Fig. 4). Data are represented as the mean +/− SEM with each data point representing one mouse. Ordinary one-way ANOVA with Dunnett’s multiple comparisons test (B-E) and two-way ANOVA with Sidak’s multiple comparisons test (F-G) *p<0.05; **p<0.01; ***p<0.001, ****p<0.0001. Statistics comparing Naive groups not shown in B-E.

Statistical analyses were performed in GraphPad Prism (San Diego, CA) and QIIME2 (56, 57). Data are represented as the mean +/− SEM with each data point representing one mouse and statistical significance set as *p* < 0.05. For these studies, we employed a variety of statistical tests, including unpaired t-test, one-way ANOVA, or two-way ANOVA. Unpaired t-tests were utilized when comparing two independent groups (e.g., XXF vs. XYF females’ number of antibody-secreting cells in Fig. 1A, where chromosome complement is the only distinguishing factor). One-way ANOVA followed by Tukey’s multiple comparisons test was utilized when comparing data across all four genotypes (e.g., *Kdm6a* expression in Fig. 2A) to allow for equal consideration of chromosome complement and gonadal sex. One-way ANOVA was followed by Dunnett’s multiple comparisons test in instances where one group serves as a reference for comparison against the other groups (e.g., % CD138 + cells “Stim” as the reference group compared with increasing concentrations of GSK J4 or GSK J5 in Fig. 3B–E). Two-way ANOVA was performed to allow for the simultaneous consideration of two independent variables while accounting for the main effects of both independent variables and their interaction. Two-way ANOVA was followed by Sidak’s multiple comparisons test (e.g., Fig. 1C), or Tukey’s multiple comparisons test (e.g., Fig. 6D) as needed, to allow for assessment of differences among multiple conditions. Statistical test used for each analysis is denoted in the figure legends. Statistical analyses of the gut microbiome bacteria alpha and beta diversities were performed in QIIME2 and utilized Kruskal–Wallis test and permutational multivariate analysis of variance, respectively.

## RESULTS

### The sex chromosome complement influences humoral responses to HKSP immunization.

To evaluate whether XX vs. XY sex chromosome complements differentially regulate sexually dimorphic humoral immune responses to HKSP immunization, the FCG mouse model was utilized (Suppl. Figure 1; (35, 36, 61)). Ovary-bearing females with XX (XXF) or XY (XYF) sex chromosomes and testes-bearing males with XX (XXM) or XY (XYM) sex chromosomes were immunized with HKSP. One-week post-immunization, the numbers of HKSP-specific IgM-antibody secreting cells (ASC) were assessed. Female and male FCG mice possessing an XX sex chromosome complement generated significantly greater numbers of HKSP-specific IgM ASC in response to immunization compared to XY mice of the same gonadal sex (Fig. 1A & D; female XXF vs. XYF *p* = 0.0267; male XXM vs. XYM *p* = 0.0272). XXF female mice also exhibited increased percentages of CD138 + plasma cells when compared with XYF female mice (Fig. 1B; *p* = 0.0050). Despite not reaching statistical significance, CD138 + plasma cell frequencies trended similarly in XXM vs. XYM males (Fig. 1E; *p* = 0.0787).

Consistent with previously published reports demonstrating enhanced immune responses against *Streptococcus pneumoniae* in females vs. males (62–64), a main effect of gonadal phenotype was identified with FCG females producing significantly higher numbers of HKSP-specific IgM ASC (mean of 567.2 ± 54–1317) in comparison to gonadal FCG males (mean of 101.7 ± 13–191; unpaired t-test *p* < 0.0001; statistical comparison not shown in Fig. 1). To examine the role of gonadal hormones in the identified XX-dependent phenotype, plasma cell frequencies were evaluated in female and male sham-operated and gonadectomized (Gdx) mice immunized with HKSP. Higher percentages of CD138 + plasma cells were observed in both intact and ovariectomized XXF vs. XYF female FCG mice (Fig. 1C; XXF vs. XYF sham *p* = 0.0039; XXF vs. XYF gonadectomized *p* = 0.0003). Males trended in a similar fashion, with greater plasma cell frequencies being observed in XXM vs. XYM mice of both sham-operated and Gdx animals (Fig. 1F). Taken together, these results demonstrate that the sex chromosome complement functions independent of circulating sex hormones to modulate immune activation in response to HKSP immunization.

#### Identification of Kdm6a as a differentially expressed gene that escapes XCI

Given that HKSP-specific immune responses were enhanced in XX vs. XY mice, we hypothesized that X-linked gene dosage effects may be important. To test this hypothesis, RNA-Seq was performed on splenocytes isolated from HKSP-immunized male and female FCG mice. As anticipated, *Xist* and *Sry* were identified as genes overexpressed in XX vs. XY cells and male vs. female cells, respectively (Suppl. Figure 3). Only two additional X-linked genes were demonstrated to be overexpressed in XX vs. XY splenocytes isolated from FCG mice immunized with HKSP (threshold of log2FC > 0.585 and FDR < 0.1): *Eif2s3x*, and *Kdm6a*. KDM6a (Lysine (K)-specific demethylase 6A, aka UTX) is a histone demethylase whose epigenetic regulatory function has previously been demonstrated to modulate other immune cells in an XX-dependent manner (65, 66), making it an interesting candidate gene for our studies. The increased expression of *Kdm6a* in XX vs. XY splenocytes (Fig. 2A; XXF vs. XYF females *p* < 0.0001; XXM vs. XYM males *p* = 0.0214) was confirmed by qRT-PCR in both total splenocytes and B cells isolated from FCG mice (Fig. 2B), and higher KDM6a protein levels (Fig. 2C–D) were detected in XX vs. XY B cells via western blot (main effect XX vs. XY sex chromosome; *p* = 0.0037, unpaired t-test).

Increased expression of an X-linked gene could indicate either biallelic expression via escape from X chromosome inactivation or increased expression from the active X chromosome. To demonstrate *Kdm6a*’s ability to escape X chromosome inactivation, RNA-FISH was performed on B cells isolated from XXF female FCG mice one-week post-HKSP immunization. The inactive X chromosome was detected using a fluorescent probe targeting *Xist*, a long, non-coding RNA that coats the inactive X chromosome resulting in its inactivation and formation of an *Xist* cloud (67, 68). Colocalization of *Xist* and *Kdm6a*-specific probes were considered indicative of *Kdm6a* being expressed from the inactive X chromosome. Approximately 13% of B cells presented with an *Xist* cloud (mean = 13%, range of 5–29%) suggesting that they were activated in response to HKSP immunization, as naïve B cells have been shown to lack an *Xist* cloud (17, 69). Of the B cells possessing *Xist* clouds, 78% (mean = 78%, range of 50–100%) exhibited colocalization of *Kdm6a* with *Xist* RNA (Fig. 2E), suggesting that *Kdm6a* is biallelically expressed.

The differential expression of *Kdm6a* was also evaluated in B cells isolated from XX vs. XY sham-operated and gonadectomized male and female mice immunized with HKSP (Fig. 2F). XX-dependent enhancement of *Kdm6a* expression was not impacted by castration of male mice (XXM vs. XYM *p* = 0.0027), and similar trends were noted in ovariectomized females (XXF vs. XYF *p* = 0.0652), suggesting that the XX vs. XY differential expression of *Kdm6a* is regulated independent of circulating sex hormones.

#### Inhibition of KDM6a ex vivo promotes plasma cell differentiation, but not in a sex chromosome-dependent manner.

We next sought to determine whether KDM6a functions to modulate plasma cell differentiation in an XX-dependent manner. Splenocytes were isolated from naïve FCG mice and stimulated with IL-4 and LPS in the presence or absence of increasing concentrations of GSK J4 or its inactive isomer GSK J5. GSK J4 is a chemical inhibitor specific for H3K27me3 demethylases, including KDM6a (70, 71). In all four genotypes (XXF, XYF, XXM, and XYM), GSK J4 (2μM) enhanced CD138 + plasma cell frequencies following *ex vivo* stimulation (Fig. 3A–E). Demonstrating the specificity of the GSK J4-mediated effect, its inactive isomer, GSK J5, did not impact plasma cell frequencies. Additionally, concentrations of total IgM in the supernatants of stimulated cells were not impacted by KDM6a inhibition (Fig. 3F–G, Suppl. Figure 4). Taken together, these data suggest that despite *Kdm6a’s* overexpression in XX vs. XY cells, KDM6a’s demethylase activity is not directly influencing CD138 + plasma cell differentiation or IgM secretion in a sex chromosome-dependent manner.

#### The gut microbiome is required for XX-specific immune enhancement.

**Figure 4:**
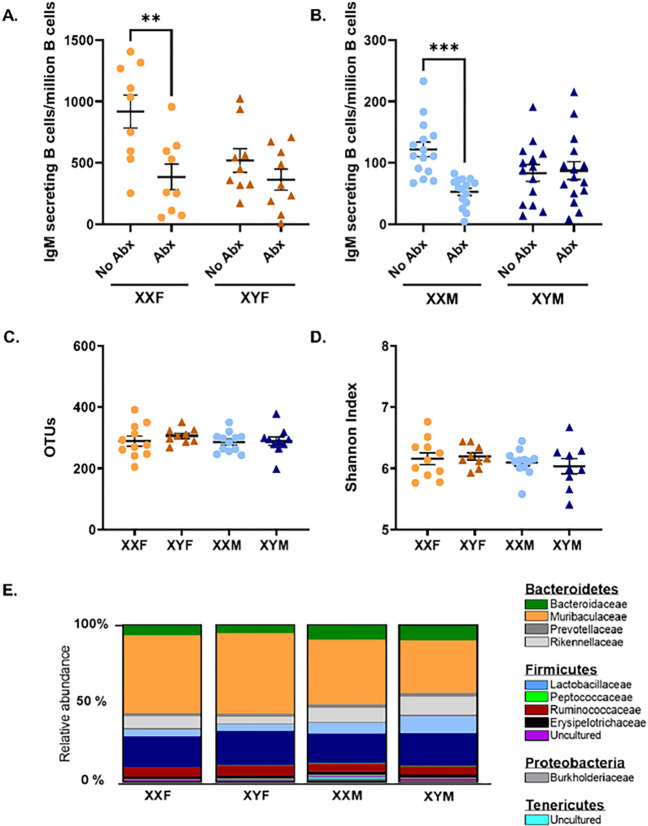
Similar gut microbiome communities influence humoral immune responses in a sex chromosome-dependent manner. The number of IgM secreting B cells produced in response to HKSP immunization was assessed by ELISPOT in male and female FCG mice possessing intact or antibiotically depleted gut microbiomes (A, B). 16s rRNA gene sequencing and metagenomic analyses were performed to assess microbiome diversity in FCG mice. The number of different OTUs as a function of the number of sequence reads (C) and Shannon diversity indexes (D) were determined. For each distinct OTU identified, percent abundancies were calculated (E). The total height of y-axis represents 100% of the assigned sequences after quality filtering, and the size of the colored regions represents proportional contributions of each phylotype shown with the top 11 families being visualized. Data are represented as the mean +/− SEM with each point representing one mouse. Statistics by two-way ANOVA with Sidak’s multiple comparisons test (A, B) or one-way ANOVA with Tukey’s multiple comparisons test (C, D) **p<0.01; ***p<0.001. Abx = antibiotics; OTUs = observable taxonomic units.

Sex biases in gut microbiome diversity have been reported and demonstrated to differentially influence immune activation (30, 72). Here, we evaluated whether the gut microbiome could differentially influence immune activation in a sex chromosome complement-dependent manner. FCG mice were administered an antibiotic (Abx) cocktail containing metronidazole (10 mg/ml), vancomycin (10 mgl/ml), neomycin (20 mg/ml) and ampicillin (20 mg/ml) via oral gavage. Control animals received sterile water alone. On day 4, mice were immunized with HKSP, and on day 10, ELISPots were performed to evaluate the number of HKSP-specific ASC (Suppl. Figure 2). Male and female XX FCG mice with intact gut microbiomes produced more HSKP-specific IgM ASC than XY mice in response to HKSP immunization. Interestingly, antibiotic administration significantly reduced XX responses (control vs. Abx; females *p* = 0.0025; males *p* = 0.0003) to levels similar to those seen in XY mice, but had no impact on XY responses (Fig. 4A–B).

**Figure 5:**
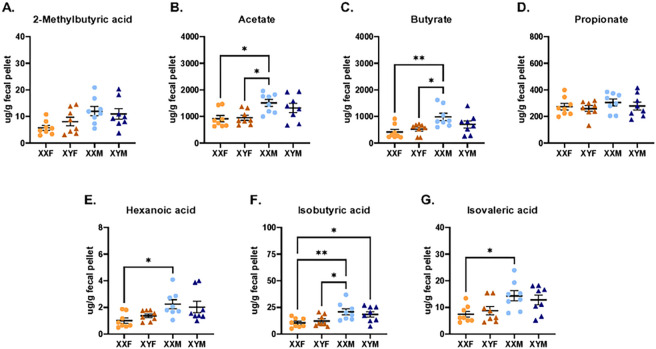
Concentrations of SCFA in the feces of male and female FCG mice. Fecal pellets from naïve FCG mice were collected and analyzed for eight short-chain fatty acids: 2-methylbutyric acid (A), acetate (B), butyrate (C), propionate (D), hexanoic acid (caproic acid, E), isobutyric acid (F), and isovaleric acid (G) by LC-MS/MS (Metabolon). Data are represented as the mean +/− SEM with each point representing one mouse. Statistics by one-way ANOVA with Tukey’s multiple comparisons test. *p<0.05; **p<0.01.

The composition of FCG mouse microbiomes was then characterized by 16s rRNA sequencing to determine if distinct microbiomes in XX vs. XY mice could explain this phenotype. Based upon calculated alpha diversity metrics, namely Shannon diversity indexes and Observed Taxonomic Units (OTUs), little diversity was identified within XXF, XYF, XXM, and XYM samples (Fig. 4C–D). Multiple beta diversity metrics were also calculated, including Jaccard, unweighted UniFrac, weighted UniFrac, and generalized UniFrac distances. While XXF female vs. XYM male microbiomes exhibited significant compositional differences based upon these metrics, beta diversity was found to be similar in XXM vs. XYM males or XXF vs. XYF females in 3 of the 4 metrics assessed (Table 3). Figure 4E represents the relative abundance of taxa identified in FCG microbiomes and emphasizes the compositional similarities between XX vs. XY animals of the same gonadal phenotype. Since compositional differences could not fully explain the microbiome-mediated enhancement of immune responses in XX mice, it was hypothesized that sex chromosome-dependent differences in concentrations of SCFAs, major metabolites of the gut microbiome, may contribute. To test this, fecal concentrations of eight distinct SCFAs, including acetate (C2), propionate (C3), and butyrate (C4), were measured using LC-MS/MS. While the concentration of individual SCFAs varied, similar concentrations were detected between XXF vs. XYF female and XXM vs. XYM male FCG mice (Fig. 5). Interestingly, male mice tended to possess higher SCFA levels than females in general.

**Figure 6:**
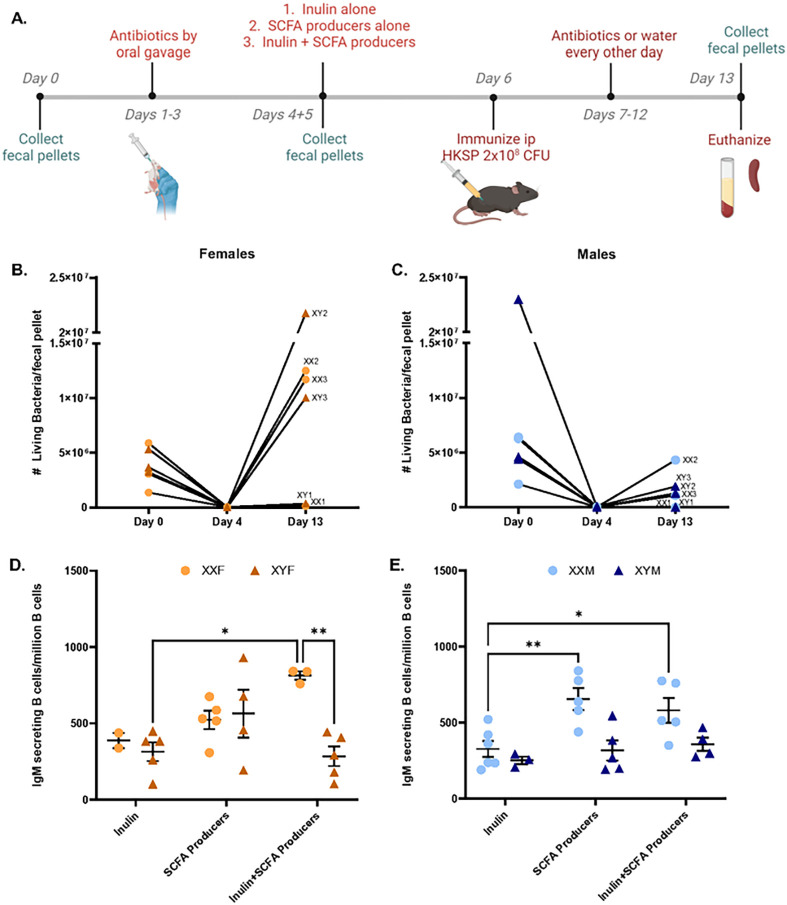
Reconstitution of gut microbiomes with SCFA-producing bacteria increased humoral responses in an XX-dependent manner. Overview of experimental design (A). Briefly, gut microbiomes of FCG mice were depleted using antibiotic oral gavage (3 days). On Day 4, mice were administered one of the following via oral gavage: inulin alone (Group 1); SCFA-producing bacteria alone (Group 2); or Inulin + SCFA-producing bacteria (Group 3). On Day 6, all mice were immunized with 2×10^8^ CFU heat-killed *Streptococcus pneumoniae*. Mice in the inulin alone exposed group received antibiotics by oral gavage every other day through Day 12, while mice receiving SCFA-producing bacteria ± inulin received water. Mice were euthanized on Day 13 and samples collected for immune response evaluations. Fecal pellets were collected on Day 0, Day 4 (prior to gavage treatments), and Day 13 to confirm colonization status. The number of living bacteria per pellet for females (B) and males (C) was assessed by flow cytometry to assess successful gut microbiome depletion and reconstitution. Immune responses were evaluated as numbers of IgM-secreting B cells in females (D) and males (E). Data point labels in B and C indicate the sex chromosome complement (XX or XY) and the group number as indicated in A. Data are represented as the mean +/− SEM with each point representing one mouse. Statistics by two-way ANOVA with Tukey’s (D) or Sidak’s (E) multiple comparisons test. *p<0.05; **p<0.01.

Despite no detected differences in XX vs. XY SCFA concentrations, it was not known if SCFAs function differently in XX vs. XY mice. Given our previous studies demonstrating that propanil enhances HKSP immune responses in an XX-dependent manner (73), we thought this was an intriguing possibility. To evaluate this potential, the endogenous gut microbiomes of male and female FCG mice were depleted using antibiotics, as described earlier. Their endogenous microbiome was then reconstituted with select SCFA-producing bacteria in the presence or absence of inulin, a fiber source that is metabolized into SCFAs. Control groups underwent antibiotic microbiome depletion, but no reconstitution (Suppl. Figure 6). All mice were then immunized with HKSP, and immune responses evaluated one week later. Live/Dead flow staining of fecal content demonstrated successful depletion of the endogenous gut microbiome and successful reconstitution in mice receiving SCFA-producers following initial antibiotic administration (Fig. 6B–C, Suppl. Figure 5). XXF females administered SCFA-producing bacteria + inulin exhibited a significant increase in HKSP-specific ASC when compared to XYF animals of the same treatment group (*p* = 0.0062), as well as in comparison to XXF and XYF microbiome-depleted controls. No increase was detected when XXF females received SCFA-producing bacteria alone. In XYF females, neither treatment (SCFA-producing bacteria + inulin or SCFA-producing bacteria alone) exhibited increased HKSP-specific responses (Fig. 6D), suggesting that SCFAs may function in an XX-dependent manner in females. However, increased HKSP-specific responses in XXM vs. XYM male mice were dependent only on the administration of the SCFA-producing bacteria, not the presence (*p* = 0.0205) or absence (*p* = 0.0025) of inulin. Similar to females, neither treatment increased responses in XY males above that of the microbiome-depleted controls (Fig. 6E).

**Figure 7:**
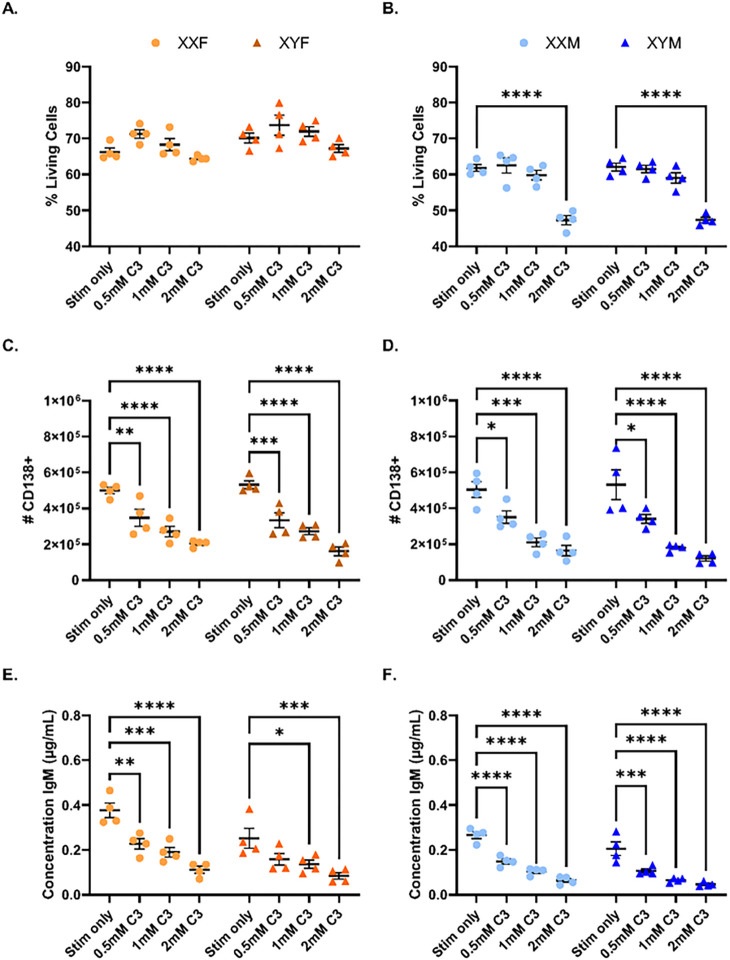
Propionate decreases plasma cell frequencies and IgM secretion similarly in all four genotypes *ex vivo*. Splenocytes isolated from naïve FCG mice were stimulated *ex vivo* with IL-4 (0.01μg/mL) and LPS (5μg/mL) in the presence or absence (Stim only) of increasing concentrations of C3 (propionate). Flow cytometric analyses evaluated cell viability (A-B) and the number of CD138+ plasma cells (C-D) 4 days post-stimulation. Supernatants were collected and total IgM concentrations were assessed by ELISA (E-F). Data are represented as the mean +/− SEM with each point representing one mouse. Statistics by two-way ANOVA with Tukey’s multiple comparisons test. *p<0.05; **p<0.01; ***p<0.001, ****p<0.0001. C3 = propionate

To further evaluate if SCFAs enhance B cell function in a sex-chromosome-dependent manner, splenocytes were isolated from FCG mice and stimulated *ex vivo* with LPS + IL-4 in the presence or absence of increasing, biologically relevant propionate (C3) concentrations. While propionate did not impact female cell viability, high concentrations (2mM) did reduce the viability of male splenocytes after 3 days of culture (Fig. 7A–B). Plasma cell differentiation was reduced in response to propionate treatment, indicated by a dose-dependent decrease of CD138 + cell numbers, as was a dose-dependent reduction in IgM secretion regardless of gonadal sex or sex chromosome complement (Fig. 7C–F).

## Discussion

In the present study, humoral immune responses against HKSP immunization were found to be differentially regulated by the presence of an XX vs. XY sex chromosome complement (Fig. 1). While the dosages of X-linked genes are typically balanced between males and females via the process of XCI (16), X-linked genes related to immunity have been demonstrated to more readily escape XCI in immune cells than other somatic cell types, and are uniquely regulated in B lymphocytes (17–21). Furthermore, the biallelic expression of multiple X-linked, immune-related genes has recently been reported to promote lymphocyte activation (15, 16, 65, 66). We therefore hypothesized that X-linked gene dosage effects may contribute to the sex chromosome-dependent phenotype we identified in response to HKSP immunization. RNA-Seq performed on splenocytes isolated from male and female FCG mice identified three X-linked genes, *Xist, Eif2s3x*, and *Kdm6a*, as being overexpressed in an XX vs. XY-dependent manner (Fig. 2A, Suppl. Figure 3). The number of potential escape genes identified was lower than anticipated given the unique regulation of XCI in B cells but was consistent with previous reports suggesting that only 3% of X-linked genes escape inactivation in mouse embryonic fibroblasts. Higher percentages, upward of 15–20%, are believed to be biallelically expressed in human cells (18, 74). While *Eif2s3x* is a commonly recognized escape gene, *Kdm6a* has only recently gained attention as an epigenetic modulator of sex-biased immune activation. Functioning as a histone H3 demethylase, *Kdm6a* has been linked to enhanced activation of female CD4 + T cell in EAE (66), as well as female microglial activation in ischemic stroke (65). Although *Kdm6a* mutations have been associated with B cell cancers (75, 76), its impact on humoral responses to infection and vaccination are only beginning to be assessed. Conservation of *Kdm6a* overexpression in XX vs. XY immune cells across species (mouse and human) (77) and its epigenetic regulatory function made it an interesting target for additional studies attempting to delineate the mechanisms that contribute to more robust humoral responses in XX vs. XY mice.

Following RNA-Seq, subsequent experiments confirmed *Kdm6a* overexpression in XX vs. XY B cells at both the RNA and protein level (Fig. 2). Since overexpression does not necessarily equate to biallelic expression, RNA-FISH was utilized to confirm *Kdm6a* expression on the inactive X chromosome. In these experiments, only 13% of the B cells isolated from HKSP immunized mice possessed detectable *Xist* clouds (data not shown), an indicator of X inactivation. Since *Xist* expression is low in naïve B cells and increases in response to both immunization *in vivo* or *ex vivo* stimulation (data not shown and (17, 69)), it was hypothesized that the B cells with detectable *Xist* clouds were reflective of activated B cell subsets (Fig. 2E). Of the B cells possessing Xist clouds, 78% demonstrated colocalization of *Kdm6a* and *Xist* signals suggesting that, similar to previous studies in mouse embryonic fibroblasts (77), *Kdm6a* is biallelically expressed by activated B cells (Fig. 2E). Due to the lack of *Xist* expression in naïve B cells, no conclusion could be drawn about whether *Kdm6a* is also biallelically expressed in this B cell subset in the current studies. However, higher *Kdm6a* gene expression levels were detected in XX vs. XY B cells regardless of their activation status (data not shown), suggesting that the biallelic expression pattern may also be retained in this subset. Small nucleotide polymorphisms (SNPs) present in the maternal vs. paternal *Kdm6a* could be utilized to confirm *Kdm6a* biallelic expression in both naïve and activated B cell subsets. However, a different background strain would be needed, as C56BL6/J mice possessed no SNPs in their *Kdm6a* genes capable of distinguishing the maternal vs. paternal X chromosome.

*Kdm6a* overexpression has been demonstrated to promote T cell (19) and NK (78) function in a sex-dependent manner. In B cell cancers, it has been identified as a tumor suppressor (79) and, in recent studies, B cell activation, isotype switching, and plasma cell differentiation were restrained by KDM6a activity (80, 81), suggesting different functional roles in different lymphocyte populations. Adding to the existing B cell data, inhibition of KDM6a activity using GSK J4 increased plasma cell differentiation in all four genotypes (XXF, XYF, XXM, XYM) at high doses following *ex vivo* stimulation (Fig. 3). Furthermore, KDM6a-mediated suppression of plasma cell differentiation was not sex chromosome-dependent, as XX and XY cells exhibited similar sensitivities to GSK J4-mediated inhibition. It should be noted that the inhibitory function of GSK J4 is not limited to KDM6a alone, but also impacts all JMJD3 histone demethylases. However, other JMJD3 histone methylase family members, including *Kdm5c*, were expressed at equivalent levels in XX and XY FCG mouse B cells (data not shown). It is possible that KDM6a influences B cell activation and differentiation in an XX-dependent manner independent of its enzymatic activity. However, this was not assessed in the current study.

A number of additional genetic factors beyond X-linked immune gene dosage effects could be contributing to the XX-dependent phenotypes described in response to HKSP immunization. Among these are genes encoded by the Y chromosome that are not expressed in XX mice (82), X gene parental imprinting, as well as the expression of X-linked microRNAs (miRNAs) (83, 84). Going beyond these traditionally studied genetic and epigenetic regulators, we propose that additional factors, including the gut microbiome, may also be influencing immune responses in an XX-dependent manner.

The gut microbiome has more recently been established as an important regulator of immunity and has been demonstrated to differentially modulate immune activation in males and females (30–32, 85). To our knowledge, the collaborative role of the gut microbiome and XX sex chromosome complement has not been evaluated. Antibiotic depletion of the gut microbiome reduced humoral responses in XX mice to levels similar to that of XY, while having no impact on the magnitude of XY responses (Fig. 4A–B), suggesting a synergistic effect. Consistent with previous reports (86–88), sex-specific differences were noted in the composition of male vs. female gut microbiomes (Fig. 4C–E). However, minimal differences were identified in the microbiome compositions of XXF vs. XYF females or XXM vs. XYM males, or in the levels of SCFAs between XX vs. XY mice of the same gonadal sex (Fig. 5). This led us to consider other mechanisms by which the gut microbiome could modulate immune responses in an XX-dependent manner. Our laboratory previously demonstrated that the immunomodulatory compound propanil enhances responses to HKSP immunization in an XX-dependent manner (34). Given that a major metabolite of propanil is also a metabolite of the gut microbiome, namely propionate, we hypothesized that propionate may likewise be capable of influencing humoral immune responses in an XX-dependent manner. In lymphocytes, the immunomodulatory effects of SCFAs have most commonly been attributed to their histone deacetylase (Class I/II) inhibitory function (40, 42, 89–92). In B cells, propionate has previously been demonstrated to inhibit HDAC activity, resulting in histone H3 hyper-acetylation, plasma cell differentiation, and class-switching (40). Subsequent opposing studies suggested that SCFAs both promote and suppress B cell responses, depending upon the exposure dose (40, 93). To our knowledge, no previous report has evaluated whether SCFAs influence immune responses differently in males vs. females or if these effects may be sex chromosome-dependent.

Here we attempted to address whether SCFAs influence humoral immunity in an XX-dependent manner in two ways. This first involved antibiotic depletion of gut bacteria and reconstitution of the microbiome with SCFA-producing species in the presence or absence of a fiber source that promotes SCFA production prior to HKSP immunization. While enhancement of XX-dependent responses required the presence of gut microbiome bacteria, this enhancement appeared to be SCFA-dependent in females only and not in males (Fig. 6D–E). Interestingly, reconstitution of the microbiomes with SCFA-producing bacteria seemed more efficient in female vs. male cells (Fig. 6B–C). These differences in reconstitution, along with differing circulating sex hormones, may explain the observed differences in females vs. males’ dependence on SCFA production. Interestingly, the main effect of gonadal sex, in which females responded more robustly to HKSP immunization than males (Fig. 1), was lost in mice possessing only select SCFA-producing bacteria. This suggests that sex-specific gut microbiome compositions influence the robustness of an immune response. However, those compositional differences alone are not mediating the XX sex chromosome-dependent effects noted following HKSP immunization, and thus requires additional study. Additionally, we tested the influence of SCFAs on XX vs. XY B cell activation in response to *ex vivo* mitogen stimulation. Using concentrations similar to those previously reported by Kim *et al*. and Sanchez *et al*., a reduction in viability was noted only at high propionate concentrations in male cells. Despite this, propionate was demonstrated to decrease mitogen-induced plasma cell differentiation in a dose-dependent manner, in concurrence with Sanchez *et al*., and it does so similarly in all four genotypes. Taken together, these data suggest that the gut microbiome influences immune responses in a multi-faceted manner, with contributions from SCFA production as well as other, not yet defined microbial components. Discrepancies between *in vivo* and *ex vivo* results may also be reflective of additional sex-specific collaborations occurring in our *in vivo* studies, which could implicate the organizational influence of sex hormones on the gut-microbiome mediated effects.

While our study provides valuable insights into the complex interactions between the sex chromosome complement and gut microbiome in shaping immune responses, it is essential to acknowledge the limitations of the FCG mouse model. Others have demonstrated similar levels of circulating sex hormones in XX vs. XY mice of the same gonadal sex (28, 94–96), but variations in gonadal morphology and function between XX and XY mice of the same gonadal sex, as well as potential differences in the phenotypic responses to cyclic ovarian hormones (97, 98), should be considered when interpreting our findings. We attempted to control for these variables by gonadectomy when possible, but the potential for hormone organizational effects prior to gonadectomy cannot be overlooked. Additionally, the variability in gut microbiome composition observed between animal facilities is a challenge whenever studying its role in biological processes. We attempted to mitigate this challenge by depleting the endogenous microbiome and reconstituting with select species, which may be a valuable strategy for obtaining reproducible results in multiple settings.

### Perspectives and Significance

While the individual impacts of sex hormones, sex chromosome complement, and the gut microbiome on immunity have been well characterized (1, 37, 99–101), the present study underscores the essential consideration that these three biological systems are intrinsically interconnected. The collaboration between sex hormones and sex chromosomes has previously been evaluated, but less is known about the interplay between these sex-specific factors and the gut microbiome. Sex hormones are known to be crucial regulators of microbiome colonization (30, 31, 85), and conversely, the gut microbiota can influence hormone production and bioactivity (32). Most previous studies investigating microbiome-dependent influences have focused on identifying sex-specific microbiome populations to explain dimorphic responses. However, in the current studies, while attempting to delineate the underlying mechanisms contributing to sex biases in immune responses to HKSP immunization, we demonstrated that similar gut microbiomes can influence immune sex biases in an XX sex chromosome-dependent manner.

## Figures and Tables

**Table 1:** *Sry, Ymt*, and *Myo* primer sequences (Invitrogen):

*Myo*	Forward: 5’-TTA CGT CCA TCG TGG ACA GCA T-3’
	Reverse: 3’-TGG GCT GGG TGT TAG TCT TAT-5’
*Sry*	Forward: 5’-AGC CCT ACA GCC ACA TGA TA-3’
	Reserve: 3’- TTG CCT GTA TGT GAT GG-5’
*Ymt*	Forward: 5’- GAG CTC TAC AGT GAT GA-3’
	Reverse: 3’-CAG TTA CCA ATC AAC ACA TCA C-5’

**Table 2:** Primer Sequences for qRT-PCR

Primer Target	Sequence
*Gapdh*	Forward: 5’-TTC ACC ACC ATG GAG AAG GC-3’
	Reverse: 3’-GGC ATG GAC TGT GGT CAT GA-5’
*Kdm6a*	Forward: 5’-TTC CTC GGA AGG TGC TAT TCA-3’
	Reverse: 3’-GAG GCT GGT TGC AGG ATT CA-5’

**Table 3:** FCG microbiome diversity: Statistical analysis of Beta-diversity indexes

Comparison	Jaccard	Unweighted UniFrac	Weighted UniFrac	Generalized UniFrac
XXF vs. XYM	0.002*	0.012*	0.020*	0.034*
XXM vs. XYM	0.038*	0.293	0.120	1.878
XXF vs. XYF	0.205	0.395	0.037*	0.068

* *p ≤* 0.05; comparison of index distances using QIIME2 plugins using PERMANOVA

## Highlights

- Humoral immune responses against HKSP immunization are influenced by the possession of an XX vs. XY sex chromosome complement.
- *Kdm6a* is biallelically expressed in XX B cells and is overexpressed in XX vs. XY-possessing splenocytes and B cells of HKSP-immunized mice.
- Inhibition of KDM6a enzymatic activity *ex vivo* promotes plasma cell differentiation, but not in in a sex chromosome-dependent manner.
- XX-dependent immune enhancement is microbiome-dependent, despite minimal differences in microbiome composition or SCFA concentrations being identified between XX vs. XY female or XX vs. XY male FCG mice.
- Reconstitution of the antibiotic-depleted gut microbiome with select SCFA-producing bacteria enhanced immune responses in XX, but not XY, FCG mice.

## Abbreviations

FCG: Four Core Genotype
HKSP: heat-killed *Streptococcus pneumoniae*
XCI: X chromosome inactivation
SCFA: short-chain fatty acids
EAE: experimental autoimmune encephalomyelitis
SLE: systemic lupus erythematosus
RNA-FISH: RNA fluorescent *in situ* hybridization
Gdx: gonadectomized
ASC: antibody secreting cell
OTUs: observed taxonomic units
